# Personal journey and perspective from psychiatric nurse and medical student to intern doctor during COVID-19

**DOI:** 10.1017/ipm.2020.39

**Published:** 2020-05-14

**Authors:** D. Lynch

**Affiliations:** Office of Prof. McNicholas, School of Medicine, University College Dublin, Dublin 4, Ireland

**Keywords:** COVID-19, Coronavirus, Medical Student, Psychiatry, Pandemic, Medical Intern, Psychiatric Nurse

## Abstract

COVID-19 or ‘Coronavirus’ has become a global pandemic since its initial report in Wuhan, China, on November 17, 2020. It is highly infectious and poses significant health risks for those in vulnerable populations. This article aims to provide perspective into an Irish experience, through the eyes of a practicing psychiatric nurse, who has recently graduated medical school and intends to work as an intern doctor.

The Coronavirus pandemic is a historical event that will have widespread effects on several generations to come. The fear of COVID-19 infection and transmission is shared universally around the globe. Although our knowledge of COVID-19 is rapidly evolving, we still have many unanswered questions. We know it now as a mutation of the SARS coronavirus, last seen in 2003, spread via respiratory droplets with initial zoonotic transmission from bats/pangolins, originating in Wuhan, China. In this article, I will provide an Irish perspective of COVID-19 from a practicing psychiatric nurse, who has just recently graduated final year medicine and intends to join the frontline of COVID-19 care as an intern doctor.

The first confirmed case of COVID-19 in Ireland was reported on February 29, 2020. Since then, its incidence rapidly increased daily. One hundred and seventy-six days since its initial presentation in Wuhan (first reported on November 17, 2019), at the time of writing (May 10, 2020, Geohive), there are 22,760 confirmed cases in Ireland, with 1,446 deaths.

COVID-19 has a mortality rate 10 times that of influenza virus, and although this is lower than its SARS counterpart, it is more infectious, largely due to its mild/asymptomatic presentation. Risk factors for mortality are age >70 years, chronic lung/cardiac conditions (including asthma), diabetes, and a compromised immune system. Those in congregated residential settings, such as nursing homes or direct-provision centers, are also at a higher risk.

At the start of the corona virus outbreak, and a few short months away from qualifying as a doctor, my inability to confidently answer questions asked by family or friends as to the likelihood of spread to Ireland, the lethality, and the virulence left me in a state of heightened personal and professional anxiety. My realization that I would soon be at the frontline further sharpened my focus.

Initially, I underestimated its potential to spread; I compared it to the SARS epidemic (2002–2003) – which had *one* confirmed case in Ireland, but no deaths – and to the swine flu pandemic (2009) – with 3000 cases and 20 deaths. This underestimation of risk was seen worldwide and likely contributed to its spread by a laxity on protective measures by individuals and/or governments.

The Irish government, while often criticised for the inadequacy of resources and leadership of our health services, should be acknowledged for its early and definitive response to managing this crisis. It splits this response into ‘three phases’ – containment, delay, and mitigation.

**Figure:**
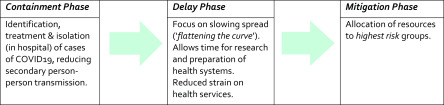

Although there is a lot of focus on the hospital management of COVID-19, with daily updates on the number of hospitalizations, those requiring ICU beds, interventions needed, etc., the larger fight against this virus occurs outside of hospitals. Some public health measures have been implemented effectively – imposing a ‘lockdown’ and encouraging 2 m social distancing – while other measures lacked clarity – creating confusion about social benefits and appropriate use of personal protective equipment (masks/gloves/goggles). The public have adapted well to new restrictions, standing 2 m apart in grocery stores and reducing non-essential travel, along with well-intentioned but misguided behaviors, such as homemade face masks and holding their breath for 30 seconds as a ‘self-test’. There has been a clear buy in from people. This became even more pronounced as the recommended quarantine, advised on March 12th, became a ‘lockdown’ on March 29th. Whether this will withstand the extended period remains to be seen. On May 1st, a 5-stage ‘roadmap’ to easing of restrictions was presented by Taoiseach Leo Varadkar; it is hoped this will provide direction to the public to ensure that they continue to adhere to restrictions, while paving a return to a functioning society and economy.

While the nation is gripped with anxiety, those with preexisting mental illness are particularly affected. This additional anxiety is justified among patients in residential care given the higher rates of ‘clusters’ – higher than expected prevalence of a disease, closely grouped in time and place. There are 382 identified clusters in nursing homes and residential centers within the Republic of Ireland as of May 7th, accounting for about half of total clusters in Ireland. These disproportionate levels in care settings *versus* the containment elsewhere in the country, as well as higher rates of mortality in these areas, have been described as ‘a national emergency’ (RTE, 2020).

Working as a psychiatric nurse in a high support residential hostel, my expectation was to see significant deterioration in the mental health of those I supported, fears of contamination, grandiose/persecutory delusions (‘I created COVID-19’ or ‘God is punishing me’), heighted anxiety, or depressive/asocial symptoms, along with non-adherence to medication and treatment reviews. Reduced contact with family and friends and reduced communal activities due to social distancing might have further exacerbated their functioning.

While anxiety is certainly heightened among the residents and more of my nursing time was spent alleviating these fears, the questions and reaction within the group were much the same as in the general population. For the most part, the residents have been open to guidance, reassured by answers, accepting of social distancing and other restrictions, engaging in regular handwashing, cough etiquette, and keeping their distance from each other and staff. There has been a clear reduction in the interpersonal difficulties between each other. My assumption of deterioration in their mental health has not been substantiated, making me consider similarities with the seminal work of Emile Durkheim in ‘Le Suicide’ (Durkheim, 1897). The directives and pleas made by clinicians and the government in Ireland’s call, where citizens were encouraged to play their part, are akin to the ‘rousing of collective sentiments’ and ‘stronger integration within society’ which Durkheim described as contributing to the reduced suicide rates at time of war (Durkheim, 1897).

It is ironic that collective ‘social distancing’ stimulates this feeling of social involvement and has similarities with Viktor Frankl’s belief that the attribution of meaning/purpose to life (no matter the circumstances) provides the ability to cope. Frankl, having lived through the holocaust, saw and shared the collective purpose created by the Jewish prisoners, which allowed them to cope through cruelties of Nazi Concentration Camps (Frankl, 1984). Although anecdotally these principles appear to apply to this pandemic, as citizens are challenged to play their part in a collective response to fighting COVID-19, mental health complaints have appeared to be reduced. It is possible that, for some, the social connectedness and sense of belonging might lead to reduced psychopathology and better engagement. However, higher rates of poor mental health have been reported in frontline healthcare workers, both in Hong Kong following the 2003 SARS pandemic (Maunder *et al*. 2003) and more recently in Wuhan (Lai *et al*. 2020). Considering this, we should prepare for possibility of a secondary ‘mental health’ pandemic in vulnerable groups such as our frontline healthcare staff.

The most challenging aspect of this pandemic from my perspective has been a lack of resources and staffing coupled with the uncertainty of working in unchartered territory. Throughout this crisis, there has been a reduction in staff numbers due to illness or annual leave. Some, with personal vulnerability such as chronic respiratory diseases, older age, or being primary carers for family members, such that the risk to themselves or their dependants was unacceptable, requested special leave. This challenge has been seen nationwide, with a public plea by the HSE (2020) to ‘Be on call for Ireland’ made to address this.

The collective anxiety about this pandemic has placed COVID-19 as a frequent differential diagnosis for many symptoms, especially respiratory or infectious. The ‘skeleton staff’ that remained needed to coordinate rapidly developed guidelines for care management in residential settings. Many staff members were not clinically trained and were given urgent education in areas such as personal protective equipment, medication administration, and monitoring of vital signs. Due to its infectious nature, staff deployed to the ‘COVID Corridor’ were often unavailable for routine or urgent duties elsewhere.

The other half of my life is as a medical student, newly graduated and eager to apply my knowledge and ‘do my part’ in fighting this pandemic. COVID-19 has had a huge impact on my final year, I went from expecting 9 weeks of clinical teaching, intern shadowing, and self-directed learning to 4 weeks of uncertainty and self-confinement, cancellation of formal exams, earlier start date, and reduced preference possibilities for intern posts. Personal goals shifted from maximizing my final grade to remembering ‘The 5 Ws of post-surgical fever’, sourcing accommodation linked to intern posting and hoping to obtain my preferential intern placements.

Commencing the internship is a daunting task at any time, commencing life as a fully fledged doctor. My cohort of classmates will be faced with caring for a disease that so many of us know so little about, the ‘COVID Experts’ having only months of clinical experience with this virus. Like many, I am scared about the unknown that I will be facing – a new job, a new city, a new virus; it can feel overwhelming. I expect to dread ‘on-calls’ and overtime and the risk of infection. I worry for my family and friends, some being ‘high-risk’ by virtue of age and illness. I also worry for my friends and colleagues, 25% of infections in Ireland have been in healthcare workers (Health Protection Surveillance Centre, 2020), and saddened by the reality that many healthcare workers have given their lives to fighting this illness.

Alongside my personal fears, I am eager to fulfill what I perceive as my national duty for my country. This pandemic has shown me that healthcare should and has transcended all borders and barriers – the international collaboration and sharing of knowledge and equipment have inspired me. The collective responses among clinicians, scientists, and industry are great examples of teamwork, reinforcing Helen Keller’s words in the face of adversity ‘Alone we can do so little; together we can do so much’.
