# Personal journey and perspective from psychiatric nurse and medical student to intern doctor during COVID-19 – CORRIGENDUM

**DOI:** 10.1017/ipm.2021.45

**Published:** 2021-09

**Authors:** D. Lynch

In the article titled ‘Personal journey and perspective from psychiatric nurse and medical student to intern doctor during COVID-19’, published in the COVID-19 themed issue in volume 37, issue 3, September 2020, a conflict of interest disclosure was omitted by the author. The author regrets this oversight.

The conflict of interest disclosure statement should have read as follows:

“The author is personally related to Prof. McNicholas, who was one of the guest editors for this special issue.”

The journal’s editorial team confirm that this conflict of interest was known to the handling editor following submission and that it was managed appropriately. They confirm that this article was subject to a thorough peer review process. Prof. Fiona McNicholas was not involved in the handling editor’s decision to accept the paper for publication. The acceptance decision was made based on the merit of the article alone.
